# Regulatory landscape and clinical implication of MBD3 in human malignant glioma

**DOI:** 10.18632/oncotarget.13173

**Published:** 2016-11-07

**Authors:** Yi Cui, Jian Li, Ling Weng, Sara E. Wirbisky, Jennifer L. Freeman, Jingping Liu, Qing Liu, Xianrui Yuan, Joseph Irudayaraj

**Affiliations:** ^1^Department of Neurosurgery, Xiangya Hospital of Central South University, Changsha, Hunan 410008, China; ^2^Department of Neurology, Xiangya Hospital of Central South University, Changsha, Hunan 410008, China; ^3^The Institute of Skull Base Surgery & Neuro-Oncology at Hunan, Xiangya Hospital, Changsha, Hunan 410008, China; ^4^School of Health Sciences, Purdue University, West Lafayette, IN 47907, USA; ^5^Biological Engineering and Bindley Bioscience Center, Purdue University, West Lafayette, IN 47907, USA

**Keywords:** glioma, epigenetics, MBD3, DNA methylation, prognostic biomarker

## Abstract

In this article we inspect the roles and functions of the methyl-CpG-binding domain protein 3 (MBD3) in human malignant glioma, to assess its potential as an epigenetic biomarker for prognosis. The regulatory effects of MBD3 on glioma transcriptome were first profiled by high-throughput microarray. Our results indicate that MBD3 is involved in both transcriptional activation and repression. Furthermore, MBD3 fine-controls a spectrum of proteins critical for cellular metabolism and proliferation, thereby contributing to an exquisite anti-glioma network. Specifically, the expression of MHC class II molecules was found to positively correlate with MBD3, which provides new insight into the immune escape of gliomagenesis. In addition, MBD3 participates in constraining a number of oncogenic non-coding RNAs whose over-activation could drive cells into excessive growth and higher malignancy. Having followed up a pilot cohort, we noted that the survival of malignant glioma patients was proportional to the content of MBD3 and 5-hydroxymethylcytosine (5hmC) in their tumor cells. The progression-free survival (PFS) and overall survival (OS) were relatively poor for patients with lower amount of MBD3 and 5hmC in the tissue biopsies. Taken together, this work enriches our understanding of the mechanistic involvement of MBD3 in malignant glioma.

## INTRODUCTION

As a deadly form of intracranial cancer, malignant glioma – the World Health Organization (WHO) grade III-IV tumors – constitutes a heavily socioeconomic burden to our healthcare system. Even with significant advancements in surgery, radiation therapy, and chemotherapy, however, the median survival for glioma patients, especially those who are diagnosed with the most aggressive glioblastoma multiforme (GBM), is extremely poor. A multitude of factors are involved in gliomagenesis, and so far considerable efforts have been invested to identify better biomarkers for patient stratification and therapeutic management. Similar to other cancers, malignant glioma is a pathological outcome resulted from genetic mutations, epigenetic aberrations, environmental stress, malfunctioned metabolism, and immunological dysfunctions [1–3]. For GBM patients received gross total resection in surgery and adjuvant treatments, the 5-year survival rate is merely 0.05% to 4.7% after the first diagnosis [4], which imperatively necessitates in-depth and comprehensive understanding of the glioma etiology.

Epigenetic regulation involves a wide range of chemical modifications that dynamically appear on chromatin elements to control gene activities without altering DNA sequences [5]. Although epigenetic aberration has been uncovered as a driving force in a variety of human diseases, its mechanistic relation to malignant glioma has been less explored. As a core epigenetic modulator, DNA methylation (mainly the addition of a methyl-group to the 5-carbon of cytosine) is able to turn off the downstream gene transcription when accumulating at the CpG islands of promoter [6]. During oncogenesis, extensive alterations in the quantity and distribution of DNA methylation have been observed [7]. Hence, the DNA methylation homeostasis is crucial to preventing cancerous transformation and under rigorous control by relevant enzymes and co-factors, including the methyl-CpG-binding domain (MBD) proteins [8]. In contrast to other MBD proteins (*e.g.*, MBD1, MBD2 and MeCP2) that can specifically associate with 5-methylcytosine (5mC) in gene repression, MBD3 has evolved to gain more functions. It has been found that MBD3 could either up-regulate or down-regulate the influential genes [9]. When incorporated into the Mi-2/NuRD complex, MBD3 can further influence nucleosome remodeling and histone acetylation [10]. In the previous work, we have discovered that MBD3 mediated a protective mechanism for controlling the proper actions of DNA methyltransferases (DNNTs) in cell cycle [11]. Altogether, the functional versatility renders MBD3 a pivotal molecule in virtually every biological process. In the light of its importance, it is of clinical significance to explore the possible deregulation and participation of MBD3 in gliomagensis.

In this study, we examine the genome-wide transcriptome regulated by MBD3 and its relevant implications to the survival of glioma patients. In glioma, the epigenetic modulation by MBD3 is bidirectional: it is connected to both active and silenced genes. By profiling the transcriptomic changes upon MBD3 knockdown (MBD3-KD) in GBM, we reveal that the genes influenced by MBD3 include a large collection of membrane receptors and transcription-related factors. Moreover, several unreported relations with regard to cancer initiation and progression have been identified. For example, our results suggest that MBD3 is required for the expression of MHC (major histocompatibility complex) class II molecules which were proposed as key factors mediating anti-glioma immunity [12–14]. We further uncover that depleting MBD3 enhances the transcription of the oncogenic miR-17-92 cluster at chromosome 13, therefore promoting tumor proliferation. In addition, a negative regulation on the long non-coding RNA (lncRNA) Gomafu (*aka* MIAT) by MBD3 was found to deregulate the alternative splicing of the tumor suppressor BRCA1 in the MBD3-KD GBM cells. Our subsequent *in vitro* and *in vivo* experiments cogently support an anti-proliferative potential of MBD3. These findings inspired us to ask whether MBD3 orchestrated a protective mechanism against gliomagenesis and could be used as a prognostic biomarker. To test this hypothesis, we recruited a clinical cohort of glioma patients (based on demographic information, clinicopathologic characteristics, and therapeutic plans), and had followed up the survival conditions for 60 months. Encouragingly, we noted that a correlated enrichment of MBD3 and 5hmC (the preferable binding site of MBD3 in brain tissues [15]) within glioma cells indicated a better PFS and OS in patients, which confers a promising basis for future large-scale clinical studies.

## RESULTS

### Loss of MBD3 expression in malignant glioma

**Figure 1:**
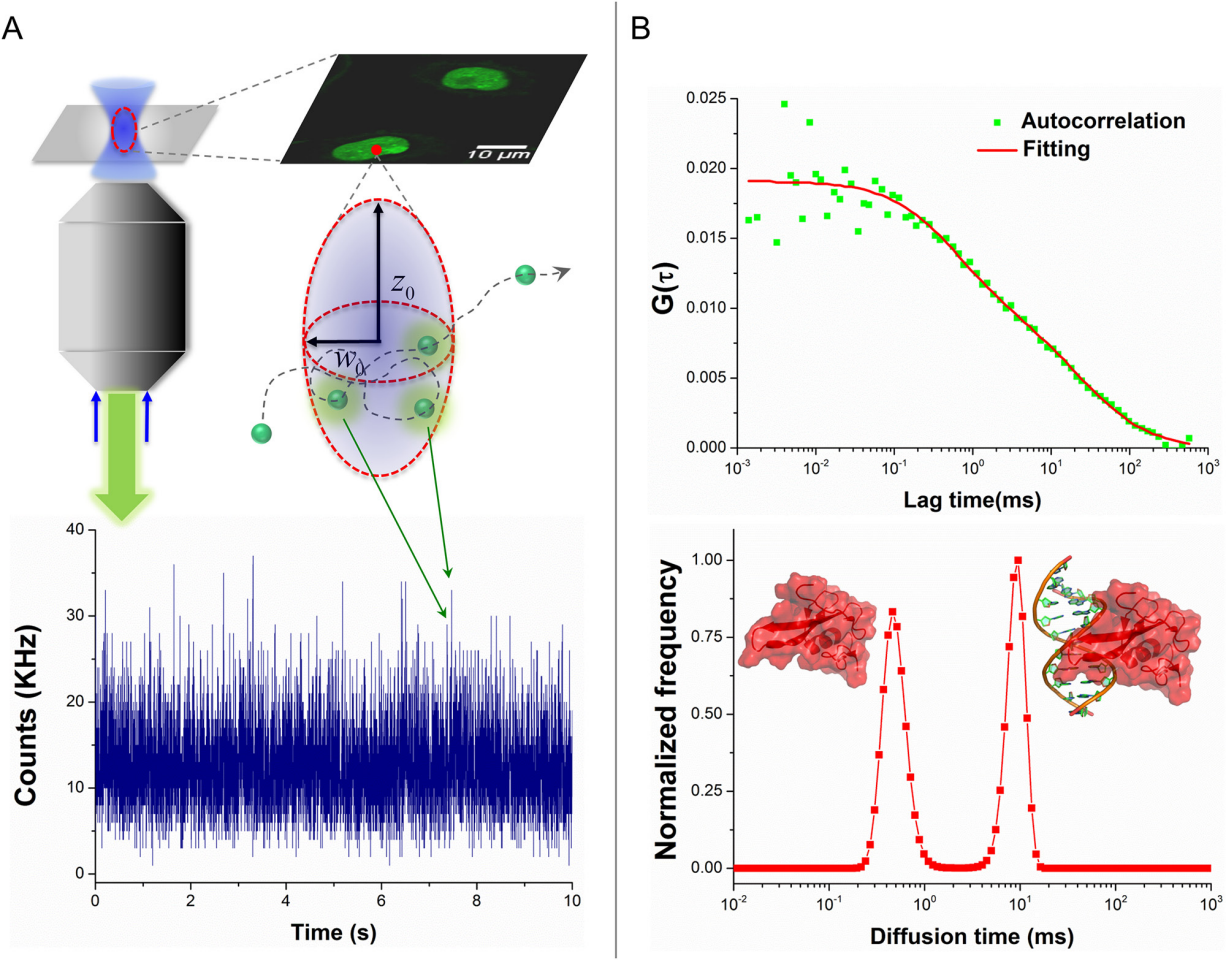
Insufficient expression of MBD3 in human glioblastoma revealed by single-molecule FCS **A.** Schematic for FCS technique to determine the intracellular diffusivity of fluorescent molecules. **B.** MBD3-GFP exhibits two distinct components of diffusivity in living SF767 cells. The component with a short diffusion time represents the free-moving proteins, while the component with a long diffusion time represents the chromatin bound proteins.

**Figure 2:**
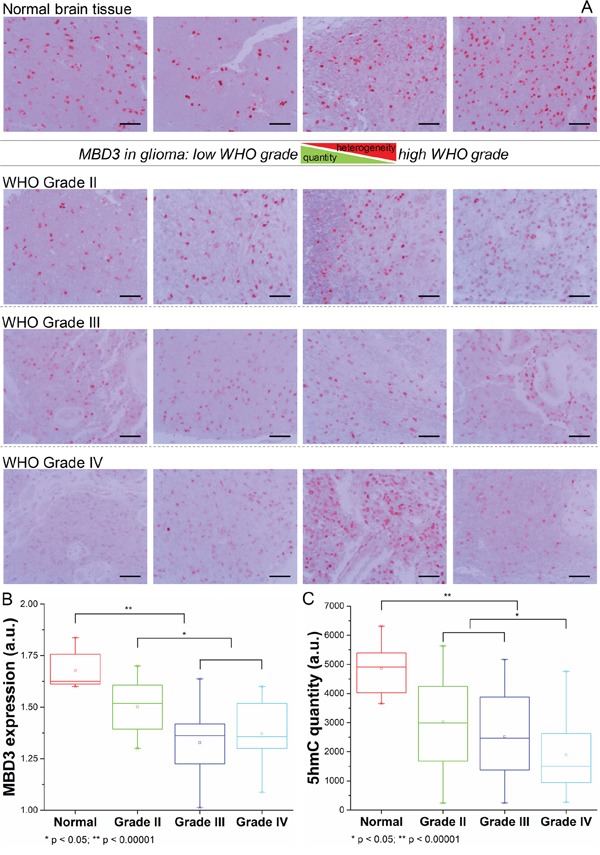
Expression pattern of MBD3 protein in normal brain tissue and malignant glioma **A.** Representative IHC images for MBD3 protein in normal brain and glioma tissues. Images were taken from an Olympus BX53 microscope fitted with a white illumination and a 20× objective lens. (Scale bars: 100 μm) **B.** Quantitative results from IHC staining (normal = 8, grade II = 16, grade III = 12, grade IV = 12, statistical tests by ANOVA). **C.** Quantities of 5hmC from the same groups of patients.

Epigenetic regulation is highly heterogeneous and varies amid different tissues, which is also reflected in the mechanism of MBD3-mediated modulation. MBD3 was elucidated to influence gene transcription through *cis* interaction with target chromatin elements. In order to assess the functional insufficiency of MBD3 in human glioma (*i.e.*, inadequate chromatin deposition), we first utilized single-molecule fluorescence correlation spectroscopy (FCS) to evaluate the biophysical occupancy of the transfected MBD3-GFP in SF767 GBM cells. In FCS, an autocorrelation function (ACF) can be derived from the rapid fluorescence fluctuation due to the molecular diffusion (Figure 1A). By fitting the ACF of MBD3-GFP with a two-component 3D diffusion model, the diffusion state of MBD3 was characterized as – the fast-moving free components (diffusion time 1-2 ms) and the slow-moving bound components (diffusion time 10-14 ms), based on which we calculated that a large proportion of MBD3-GFP (> 50%) could incorporate into the glioma epigenome (Figure 1B). This set of experiments indicates a non-redundancy or insufficient expression of endogenous MBD3 in human glioma, which gives rise to the binding space for exogenous MBD3 molecules. Following this line of findings, a negative correlation between the WHO grade of glioma and the MBD3 abundance was further revealed (Figures 2A-2B, S10). Interestingly, the expression of MBD3 shares the reducing pattern with the 5hmC quantity in high-grade glioma (Figure 2C). Hence, we proposed that the deregulated MBD3 may join other oncogenic events (*e.g.*, global DNA hypohydroxymethylation) to promote gliomagenesis and metastasis.

### Regulatory landscape of MBD3 in malignant glioma

**Figure 3:**
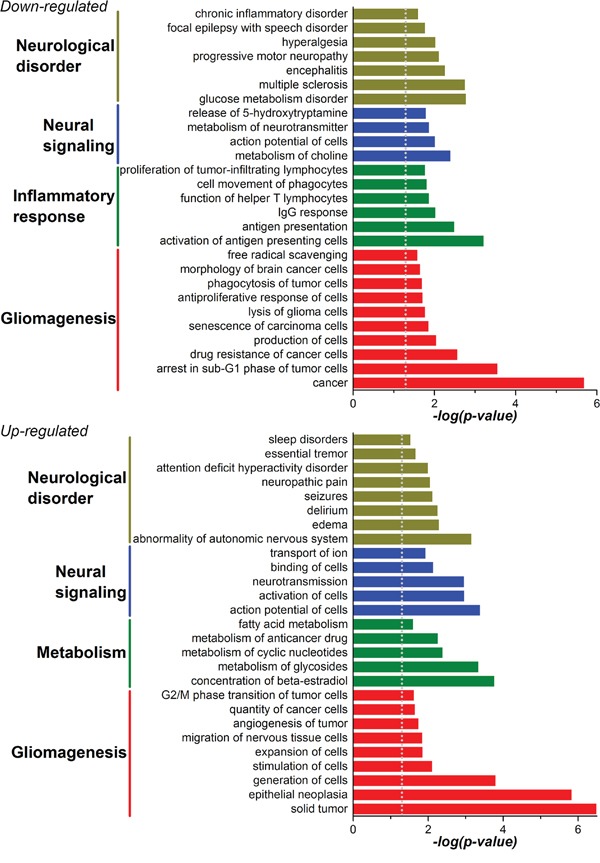
Cellular functions and diseases related to the DEGs in MBD3-KD GBM cells were profiled with gene enrichment analysis in the IPA software

Progression to higher malignancy is a pathologic tendency during glioma recurrence, while the detailed mechanisms are largely unknown. Considering the serial loss of MBD3 in the process of gliomagenesis, we attempted to profile the key genes influenced by MBD3. A set of siRNAs targeting MBD3 was introduced into SF767 cells and the knockdown efficiency was confirmed by quantitative real-time PCR (qRT-PCR), western blot, and immunofluorescence (Figures S1A-S1B). Then the genome-wide changes of transcriptome were determined by high-throughput microarray (Figure S1C). In parallel, the DEGs between low-grade glioma and GBM (in biopsy samples) were screened (Figure S2). Hence two sets of DEGs were obtained and the encoded proteins were compared. As shown in Figures S3-S4, a considerable overlap in the impacted protein functions between these two sets of DEGs is noted, which implicates that during the progression from low-grade to high-grade gliomas, MBD3 is involved in a substantial portion of gliomagenetic pathways and functions. The top-matched gene ontology (GO) term in the down-regulated DEGs by MBD3-KD is the “MHC II protein complex” – a surface protein complex presenting antigens to the CD4+ T cells (Figure S3). In analysis of the up-regulated DEGs, the major differences between low-grade glioma and GBM are “nucleic acid binding proteins” and “transcription factors”, while depleting MBD3 further disturbs the expression of some “receptor proteins” (*e.g.*, G-protein coupled receptors) in GBM cells (Figure S4). Other up-regulated DEGs by MBD3-KD include transport proteins (*e.g.*, ion channel proteins and solute carrier family), cytochrome P450 family, growth factors (*e.g.*, FGF and IGF), and dynein proteins, all of which are hallmarks for active cell metabolism and proliferation. Moreover, the MBD3-KD induced DEG-dependent cell functions and diseases, as revealed by Ingenuity Pathway Analysis (IPA), mainly point to “gliomagenesis”, “inflammatory responses”, “metabolism”, and “signaling” in the central nervous system (CNS) (Figure 3). Out of the 7,419 probed lncRNAs, about 600 are determined to be significantly changed upon MBD3 knockdown, though most of their functions require future exploration due to the lack of well-defined annotations. One interesting finding is that the lncRNAs regulated by MBD3 does not follow the expected distribution on some chromosomes. Instead, a skewed distribution is observed (Figure S5). Chromosomes 2, 5, 6, 8, 13, and X encompass a higher percentage of MBD3-influenced lncRNAs than the expected distribution of lncRNAs. Given the regulatory importance of lncRNAs to the chromatin integrity, transcription rate, and post-translational modification, our results preliminarily suggest that the dysfunction of MBD3 may compromise more functions relating to these specific chromosomes.

### Correlation between MBD3 and MHC class II molecules

**Figure 4:**
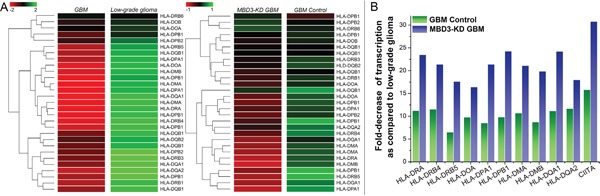
MBD3 facilitates the basal expression of MHC class II molecules in GBM cells **A.** Raw signal readings from microarray are shown. For genes having multiple probes in the array, transcription level was averaged. **B.** The MBD3-regulated HLA genes in GBM and MBD3-KD GBM cells were normalized to the quantities in low-grade glioma.

Since the down-regulation of MHC II protein complex was strongly indicated by GO analysis, we extracted the raw data to inspect the detailed transcription of each subunit composing this complex (Figure 4A). MHC class II protein complex is a heterodimer comprising α chain and β chain, transcribed from the HLA genes on chromosome 6. Five major HLA-encoding regions exist: HLA-DM, HLA-DO, HLA-DP, HLA-DQ, and HLA-DR. It has been demonstrated that MHC class II molecules could be constitutively expressed in glioma cells and their level is negatively associated with the tumor invasion and malignancy [13, 16, 17]. As shown in Figure 4B, in comparison with low-grade glioma, GBM has a significantly lower transcription in almost all of the HLA components. With MBD3 knocked down, ten HLA variants experienced a further reduction in GBM cells. We hypothesized that one intermediate mechanism accounting for this MBD3-induced massive modulation was through the MHC class II transactivator (CIITA) gene as it exhibited the same reduction trend in the array results. Transcription of CIITA is subjected to epigenetic inactivation, especially by DNA methylation in the promoter IV region [16, 18, 19]. MBD3, in the context of Mi-2/NuRD complex, is involved in maintenance of a hypomethylated state at its occupancy site and thus can facilitate gene expression. In the MBD3-KD GBM cells, a global DNA hypermethylation was accordingly noted (Figure S6A), which is consistent with our previous study [11]. With methylation-specific PCR (MSP), the methylation level of the CIITA promoter IV was also found to rapidly increase by ~20% after 72 hours of MBD3-KD (Figure S6B), which supports an epigenetic control on the CIITA gene expression by MBD3. Intriguingly, the human HLA gene complex resides on chromosome 6 where MBD3 preferably influences the transcription of lncRNAs, which would bring about new insights regarding the interconnection among epigenetic modifications, regulatory lncRNAs, and immune functions.

### Influence of MBD3 on lncRNAs miR-17-92 cluster and Gomafu

**Figure 5:**
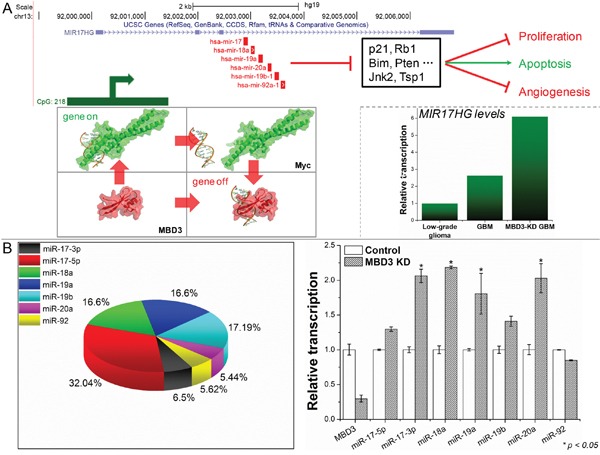
MBD3 fine-tunes the Myc-mediated transcription of MIR17HG **A.** The transcriptional regulation and main downstream effectors of MIR17HG are presented (upper panel); and the pri-miRNA levels in low-grade glioma, GBM, and MBD3-KD GBM were probed by microarray. **B.** The composition of mature miRNAs from MIR17HG in GBM cells and their level changes upon depleting MBD3 were quantified by qRT-PCR (n = 3, mean ± s.d.).

The miR-17-92 cluster is among the first characterized microRNAs (miRNAs) underpinning a variety of molecular events to promote an oncogenic process. From this polycistronic locus, a precursor transcript (pri-miRNA) is encoded and can yield six mature miRNAs: miR-17 (further derive the mature miR-17-5p and passenger miR-17-3p), miR-18a, miR-19a, miR-20a, miR-19b-1 and miR-92a-1 [20]. These miRNAs individually or together regulate a broad spectrum of genes including CDKN1A (p21) and PTEN, and thus impact the cell fate (Figure 5A) [21, 22]. Mainly activated by Myc and E2F proteins, increased expression of miR-17-92 has been reported in a number of malignancies [23, 24]. From the microarray, the probed transcript representing the miR-17-92 cluster, namely MIR17HG, in GBM is significantly higher than that in low-grade glioma. We also noticed that the level of MIR17HG would experience a further increase when MBD3 was suppressed. Recent research has revealed that MBD3/NuRD could directly associate with c-Myc to repress the downstream transcription from the c-Myc targeted genes [25], which therefore supports the regulatory correlation between MBD3 and MIR17HG. Specifically, we quantified each mature miR-17-92 miRNAs and noted that miR-17-3p, miR-18a, miR-19a and miR-20a experienced the most significant increase (Figure 5B). To validate, qRT-PCR and flow cytometry were conducted: upon the MBD3-KD mediated transactivation of miR-17-92, p21 was substantially reduced, resulting in aberrant transition in the G2/M of cell cycle (Figure S7).

In the MBD3-KD GBM cells, another significantly influenced lncRNA is Gomafu (increased by 12.75 fold), which is a 9-10 kb intra-nuclear transcript that can bind to the splicing factor 1 (SF1) to inhibit alternative splicing of mRNAs [26]. A typical gene undergoing frequent splicing is BRCA1 whose full-length mRNA contains 24 exons and can generate more than 10 variants through alternative splicing [27]. Among all the variants, the full-length (WT), Δ(9, 10), Δ(11q), and Δ(9, 10, 11q) are four predominant BRCA1 gene products [28]. The Δ(11q) and Δ(9, 10, 11q) variants respectively give rise to the functional isoforms of BRCA1a and BRCA1b proteins [29, 30]. In light of this knowledge, we next evaluated the quantities of these four major BRCA1 variants after MBD3 knockdown. From the qRT-PCR results, it is apparent that both the Δ(11q) and Δ(9, 10, 11q) decreased while the BRCA1 WT increased (Figure S8). In contrast, the Δ(9, 10) did not show a substantial change. Hence, we speculate that Gomafu tends to suppress the splicing-out of large exons from BRCA1 because exon.11q (3,308 bp) is much longer than other exons. As a result, the disturbed equilibrium in BRCA1 expression would exacerbate the glioma progression, as supported by the Kaplan-Meier survival data in the NCI REMBRANDT database (https://caintegrator.nci.nih.gov/rembrandt, Figure S9) [31].

### Clinical implication of MBD3 expression in glioma prognosis

**Figure 6:**
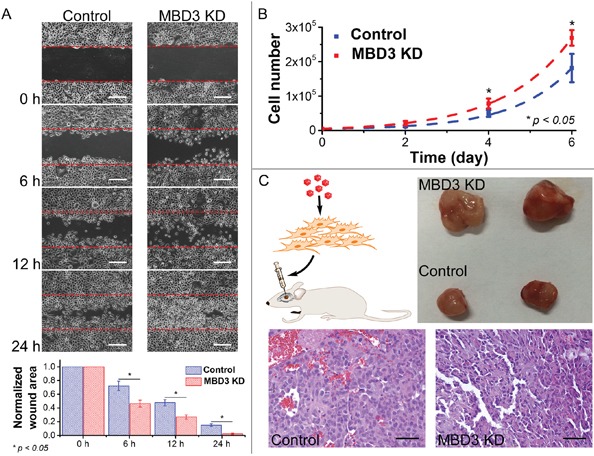
Influence of MBD3 on glioma migration and proliferation **A.** Migration assay was performed using glioblastoma cells with and without MBD3 siRNA (n = 3, mean ± s.d.). (Scale bars: 100 μm) **B.** Proliferation rates of glioblastoma cells with and without MBD3 siRNA (n = 4, mean ± s.d.). **C.** *In vivo* tumor formation assay was performed by injecting glioblastoma cells into mouse brain. By H&E staining, the MBD3-KD GBM cells show more pleomorphic and basophilic nuclei. (Scale bars: 100 μm).

**Figure 7:**
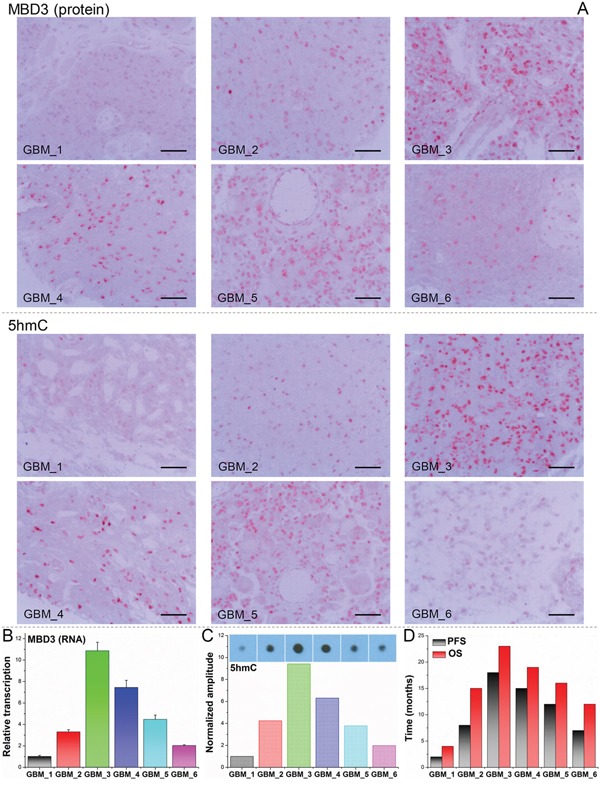
Dual-quantification of MBD3 and 5hmC exerts a promising prognostic power for GBM patients 6 GBM patients with correlated MBD3 and 5hmC content were followed up after surgery. **A.** IHC staining for MBD3 protein and 5hmC. (Scale bars: 100 μm) **B.** qRT-PCR for MBD3 mRNA. **C.** Dot blot for 5hmC. **D.** Survival times of the patients. PFS: progression-free survival; OS: overall survival.

**Figure 8:**
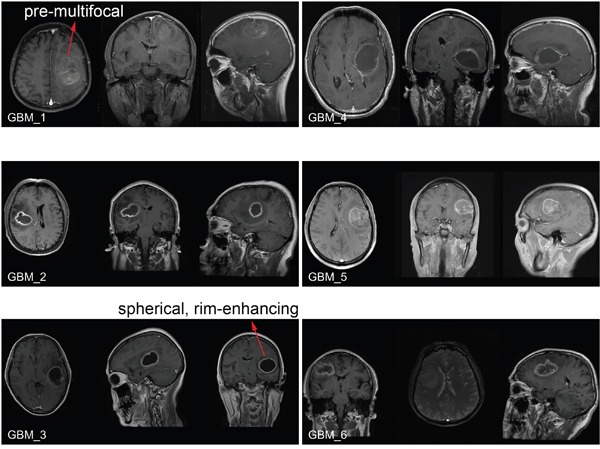
Brain MRI images of GBM patients enrolled in the clinical cohort Before surgery, the tumor growth was examined by the T1 post-contrast MRI imaging. The pre-multifocal growth of MBD3-low GBM features a rapid tumor expansion, aggressive infiltration to the surrounding normal tissue, and poor survival. In comparison, the spherical, rim-enhancing MBD3-high GBM features a relatively mild growth, confined edge, and better survival.

Following the experiments at the molecular and cellular levels, we explored the possibility of using MBD3 as a potential prognostic biomarker due to its control on glioma migration and proliferation (Figure 6). To test this hypothesis, a pilot clinical cohort was designed and the enrolled glioma patients were strictly selected by matching their demographic information, pathological characteristics, surgical outcomes, and adjuvant therapies. In normal brain tissue, the abundance of MBD3 shows a strong signal from IHC staining. In malignant glioma, MBD3 is also prevalently expressed but the average level negatively correlates with the WHO grade. Intriguingly, the quantity of MBD3 exhibits a significant heterogeneity among the high-grade tumors, particularly apparent in grades III-IV (Figure 2B), which inspired us to check whether a difference in patients’ survival existed. Besides protein, the MBD3 mRNA extracted from clinical samples was quantified by qRT-PCR (Figure S11); however, no direct correlation was noted between the MBD3 transcripts and glioma grade, implicating a decreased efficiency of mRNA translation or an increased rate of protein degradation in high-grade gliomas. 5hmC has been discovered as a major DNA modification in association with MBD3 and some efforts aimed to exploit the clinical implication of 5hmC in glioma have been attempted [32–34]. The reduction of 5hmC in high-grade glioma was indeed noted in our study (Figure 2C). It is well established that epigenetic modifications and associated proteins function in a cooperative manner. As the DNA modification *per se* is not convenient to quantify, it is rational to exploit some relevant epigenetic proteins as the alternative in clinical applications. In this regard, a group of patients bearing correlated levels of MBD3 and 5hmC in tumor biopsies were recruited. In the 60-month follow-up, MBD3 – as well as 5hmC – exhibited a positive correlation with the patients’ survival. The PFS of GBM patients who received gross total resection in surgery and adjuvant therapies (*i.e.*, radiotherapy and temozolomide) was highly proportional to the intra-tumor levels of MBD3 and 5hmC (Figure 7). This correlation was also observed in the WHO grades II-III patients (Figures S12-S13). From MRI imaging, tumors with a relatively high abundance of MBD3 and 5hmC showed a more confined growth whereas low abundance of MBD3 and 5hmC contributed to a more aggressive, pre-multifocal growth (Figure 8). Therefore, this set of data supports the potential of MBD3 as a novel epigenetic marker to complement 5hmC in the clinical management of malignant glioma.

## DISCUSSION

Although malignant glioma is a prevalent form of primary brain tumor leading to devastating outcomes, research on involvement of epigenetic proteins in gliomagenesis is still sparse. Like other solid tumors, glioma also manifests a plethora of epigenetic aberrations, but it is far from clear if the associated proteins of DNA methylation play a role in this pathology since some early efforts were attempted [35, 36]. In contrast to other MBD proteins, human MBD3 exhibits more elusive and dynamic interaction with methylation sites [37]. For instance, MBD3 has long been thought of losing its binding specificity to 5mC; however, the co-localization between MBD3 and methylation marks has been unambiguously detected in a myriad of studies [9, 11, 38–41]. On the other hand, overexpression of MBD3 could induce DNA demethylation [42], implying an exquisite participation of MBD3 in the DNA methylation homeostasis. In addition to being a DNA-binding protein, MBD3 also serves as a core subunit of the Mi-2/NuRD complex which modulates histone deacetylation and nucleosome remodeling [10, 43]. MBD3 has been proven to be a key factor in inhibiting tumorigenesis and naïve pluripotency in recent studies [25, 44–46]. In spite of some research discrepancies, the functional importance of MBD3 to the CNS is evident, spanning from cell pluripotency to differentiation, synaptic formation to lineage commitment [15, 47–49]. This study further suggests a MBD3-mediated anti-glioma network. Consistent with the literature [9], we characterize that MBD3 exerts influences on both active and silenced genes. Our bioinformatics and experiment data supports that maintenance of the MBD3 abundance might herald a better prognosis for glioma patients.

The regulation on MHC class II molecules by MBD3 is one of the major findings here because it will provide us with ample avenues to understand the immune escape of gliomagenesis and devise targeted immunotherapies. For a considerably long period, human brain was thought to own an immune privilege due to the existence of blood-brain barrier (BBB) and the absence of intracranial lymphatic structures [50], which could facilitate the CNS tumors to evade immune surveillance. However, the integrity of BBB in glioma is often sabotaged by tumor invasion and immature vasculature, and the antigen presentation-required MHC molecules are found in a variety of glial cells, including microglial cells, astrocytes, and even glioma cells. All these phenomena at least indicate an anatomic connection between the CNS and immune system. The very recent discoveries of glioma infiltrating lymphocytes and meningeal lymphatic vessels validate the participation of immune functions in restricting the initiation and progression of malignant glioma [51, 52]. Enhancing the presentation of tumor antigens has been proposed as a viable therapy option, and peptide-based glioma vaccine was successfully developed to activate the MHC class II molecules-dependent CD4+ T cells [14]. However, this approach is practically limited because down-regulation of MHC molecules is a pathological characteristic of malignant glioma [13]. In this regard, restoring the expression of MHC molecules becomes a key in immunotherapies against glioma. In several early attempts, *in situ* delivery of interferon-gamma (IFN-γ) by viral vectors was applied to enforce the MHC class II expression in glioma cells and enhance the anti-tumor activity of T cells [53, 54]. From our results, the expression of MHC class II molecules was found to be utterly low in GBM compared with that in normal brain tissue and low-grade gliomas. Nevertheless, by epigenetically regulating the CIITA gene and relevant lncRNAs on chromosome 6, MBD3 may be able to reactivate MHC class II molecules and hence serve as a therapeutic target to advance the current wave of immunotherapies. We anticipate that glioma tissues with relatively more MBD3 would respond better to therapeutic vaccination and immune-checkpoint-blockade agents (*e.g.*, PD-1/PD-1L inhibitors and CTLA-4 blockade). Moreover, synthetic biology tools to boost MBD3 expression hold certain promise in future gene therapies.

Upon targeting a short “seed” region (6-8 nt) in mRNAs to halt translation, miRNAs provide another layer of post-transcriptional regulation on gene expression. A growing body of reports has supported the involvement of miRNAs in various stages of oncogenesis. The miR-17-92 cluster, also dubbed “oncomiR-1”, gives birth to a group of mature miRNAs involved in cell proliferation, apoptosis, and angiogenesis. Transcription from the miR-17-92 cluster (*i.e.*, the MIR17HG on *C13orf25*) is mainly activated by two classes of transcription factors – the Myc and E2F proteins. Loss-of-control on transactivation of miR-17-92 has been identified as a hallmark in hematopoietic malignancies and solid tumors. For instance, overexpression of miR-17-92 was reported in neuroblastoma and correlated with poor patient prognosis [55–57]. Other miR-17-92 driven malignancies include lymphoma [58], leukemia [59], retinoblastoma [60], medulloblastoma [61], colorectal cancer [62], breast cancer [63] and so on. In malignant glioma, the copy-number amplification of MIR17HG in genome is rare, indicating its overactive transcription by Myc or E2F. In our study, the up-regulation of miR-17-92 in GBM rather than in low-grade gliomas, can be primarily attributed to the significantly higher expression of n-Myc (up by 4.46-fold) and E2Fs (Figure S14). As a control mechanism, MBD3 and its associated Mi-2/NuRD complex could epigenetically fine-tune the transactivation activity of Myc proteins [25]. This partially explains the observed increase of MIR17HG in GBM cells when MBD3 was depleted. Consequently, excessive mature “oncomiR-1” was produced and impacted a series of molecular pathways, such as the p21-mediated cell cycle progression (Figure S7).

Gomafu is a CNS abundant lncRNA that retains its nuclear localization after transcription. One characterized function of Gomafu is to suppress the alternative splicing of pre-mRNAs through sequestration of SF1 and other nuclear splicing factors [26]. This mechanism is implicated in brain development [64], post-mitotic neuronal function [65], addiction [66], and schizophrenia [67]. In our microarray results, the expression of Gomafu was negatively controlled by MBD3. Following this clue, we were tentative to correlate MBD3 with the alternative splicing of BRCA1 in GBM cells. Although BRCA1 is a tumor suppressor gene responding to DNA damage, the disequilibrium among its spliced variants may adversely abrogate the therapeutic benefits from the DNA damage-inducing agents, such as temozolomide and platinum drugs [68, 69]. With an over-activation of Gomafu by depleting MBD3, the full-length BRCA1 increases at the cost of two major spliced variants BRCA1a and BRCA1b. Even so, we cannot rule out the impacts from other MBD3-KD induced epigenetic aberrations, such as disturbed gene body methylation or histone modifications [70, 71], on the alternative splicing of BRCA1. Clinically, the altered ratio of BRCA1 variants would undermine the survival of glioma patients who need standard temozolomide treatment.

We show that MBD3 can complement 5hmC in prediction of glioma prognosis. This positive correlation inspired us to explore the underlying implications. Catalyzed by the ten-eleven translocation (TET) family enzymes, 5hmC is the first oxidization product of 5mC [72]. 5hmC is heterogeneously distributed among different tissues, while the CNS harbors a rich content of this cytosine mark (0.3-0.7% of the whole genome) [73, 74]. 5hmC locates in the midst of the DNA demethylation pathway, indicating that is critical for dynamic gene activation. Other than being a demethylation intermediate, 5hmC has been proposed as an independent epigenetic modification which regulates a plethora of biological functions through interactions with distinct binding proteins [39]. MBD3 is one of the few factors capable of associating with 5hmC [15, 38], though some research disparities challenge the specificity of the MBD3-5hmC interaction. Considering the context-dependent distribution, time-dependent abundance, and extensive cooperation of epigenetic modifications, the connection between MBD3 and 5hmC in human CNS should be substantive because the highly enriched 5hmC needs to be interpreted by appropriate readers. Interestingly, the enrichment of MBD3 or the 5mC-binding protein MBD2 indicates entirely opposite outcomes in the glioma survival even though they share a >70% similarity in protein sequences (Figures S15-S16). This intra-family divergence highlights the regulatory intricacy of epigenetic mechanisms and signifies the MBD3-MBD2 contention in the DNA methylation homeostasis as discovered before [11, 75]. The medical significance about a reduced 5hmC level in various cancers, including malignant glioma, has been preliminarily established. In this study, we incorporate one of the 5hmC-associated factors to strengthen the reliability of using epigenetic markers to stratify the glioma survival, and provide an epigenetic biomarker-based alternate strategy to facilitate pathological diagnosis. In the long term, we expect this approach to be useful not only for clinical management of individual patients but also for epidemiological design of large-scale studies.

## MATERIALS AND METHODS

### Ethics statement

Investigation regarding patients and animals has been conducted in accordance with all ethical standards, following the Declaration of Helsinki as well as the national and international guidelines. This study has been approved by the Ethic Committee of Xiangya Hospital of Central South University, Changsha, China.

### Cell culture and siRNA transfection

Human GBM cell line SF767 was routinely cultured in Iscove's Modified Dulbecco's Medium (IMDM) supplemented with 10% fetal bovine serum, 1% PenStrep antibiotics and 1% glutamate. After revival from liquid nitrogen, cells sub-cultured within 4 to 6 passages were used for knockdown experiments. The specific siRNAs targeting human MBD3 mRNA and the scramble control siRNAs were obtained from the Santa Cruz Biotechnology. Transfection was performed with the siRNA Reagent System (sc-45064). After 72 hours of incubation, the knockdown efficiency was determined by qRT-PCR, western blot, and immunofluorescence staining. From the quantification results, a 70-75% of decrease in MBD3 transcription can be maintained up to 6 days after siRNA transfection, based on which microarray, proliferation assay and migration assay were performed.

### RNA microarray

The difference of whole-genome transcriptome between low-grade glioma and GBM, as well as the DEGs before and after knocking down MBD3 in human SF767 GBM cells were investigated by the SurePrint G3 Human Gene Expression 8 × 60K Microarray Kit (Design ID: 028004, Agilent) that covers 27,958 Entrez Gene RNAs and 7,419 lncRNAs. Four biological replicates/samples for each condition were prepared. After RNA extraction by TRIzol reagent (Invitrogen, for biopsy tissues) or RNeasy Mini Kit (Qiagen, for cultured cells), cDNA and Cy3-labeled cRNA were sequentially synthesized with LowInput QuickAmp Labeling Kit (Agilent). The hybridization step lasted for 17 hours at 65°C. After mild wash, the array slides were imaged by a SureScan scanner followed by feature extraction. The hybridization quality was assessed by monitoring the spike-in signals of ERCC and E1A control probes. The raw intensity readout within the array was normalized to the intensity value of the 75th percentile of all non-control probes in the array. For genes having multiple probes, the expression level was mathematically averaged. Approximate 2,000 DEGs based on fold-change and *p*-value (fold-change > 3 and *p*-value < 0.05 for clinical samples; fold-change > 1.5 and *p*-value < 0.1 for cultured cells) were determined with moderated *t-*test in GeneSpring GX software. Protein function classification was conducted with the online PANTHER classification system (http://www.pantherdb.org) [76, 77]. GO analysis was performed by GeneSpring GX. Analysis for the DEGs-involved cellular functions/diseases was performed with IPA software package (http://www.ingenuity.com/products/ipa). The information of differentially expressed lncRNAs was manually annotated and cross-verified with referring to:

EMBL-EBI database (http://www.ebi.ac.uk)

long non-coding RNA database (http://lncrnadb.com)

miRBase (http://www.mirbase.org)

human lincRNA catalog database (http://www.broadinstitute.org/genome_bio/human_lincrnas)

The complete microarray raw data and expression documents have been deposited to the NCBI's Gene Expression Omnibus and are accessible through the GEO Series accession number GSE79878 (http://www.ncbi.nlm.nih.gov/geo/query/acc.cgi?acc=GSE79878).

### qRT-PCR

RNA was extracted and purified from SF767 cells with RNeasy Mini Kit (Qiagen) followed by reverse transcription with iScript cDNA synthesis kit (Bio-Rad), according to the manufacturers’ instructions, respectively. PCR amplifications for BRCA1 variants, p53, p21 and Caspase3 were performed in a StepOnePlus system (Applied Biosystems) with SYBR Green PCR Master Mix (Life Technologies). *ΔΔCt* method was used to normalize all the transcription level to the internal control gene GAPDH. All the primers information is provided in Table S3. The mature miRNAs transcribed from the miR-17-92 cluster were quantified with a real-time PCR assay kit (Signosis). After RNA extraction, the target miRNAs were separated by ligation and magnetic beads. U6 non-coding RNA was used as the internal control.

### Dot blot for 5hmC in clinical samples

Genomic DNA from brain or tumor tissues was isolated by cell lysis buffer (100 mM Tris-HCl, pH 8.5, 5 mM EDTA, 0.2% SDS, 200 mM NaCl, 0.667 μg/μl proteinase K) at 55°C overnight. In the second day, the samples were added with an equal volume of phenol:chloroform:isoamyl alcohol (25:24:1 saturated with 10 mM Tris, pH 8.0, 1 mM EDTA), mixed completely, and centrifuged for 5 minutes at 14,000 g. The aqueous layer solution was transferred into a new Eppendorf tube and precipitated with equal volume of 100% isopropanol. The genomic DNA was recovered and dissolved with 10 mM Tris-HCl (pH 8.0). Genomic DNA samples were further sonicated into 500 bp by a Misonix sonicator 3000 system. The concentration of sonicated DNA was determined by Nano-Drop 1000 (Thermo Scientific). The sonicated genomic DNA was denatured in TE buffer with 0.4 N NaOH at 100°C for 10 minutes, immediately chilled on ice for 10 minutes, and spotted onto a positively charged nylon transfer membrane (GE Healthcare) with a Bio-Dot Apparatus (Bio-Rad). The membrane was baked for 30 minutes at 80°C and then blocked with PBS containing 5% dry milk and 0.1% Triton X-100 at room temperature for 1 hour. The primary rabbit anti-5hmC antibody (1:10,000, Active Motif) was applied to the membrane and incubated at 4°C overnight. Next day, the membrane was rinsed with fresh PBS and the signal was developed after incubation with HRP-conjugated secondary antibody for 30 minutes. The final signal was visualized with an ECL-Plus system (Amersham Pharmacia Biotech).

### Immunohistochemistry for MBD3 and 5hmC in clinical samples

5 μm-thick tissue slices were sectioned from formalin-fixed paraffin-embedded surgically resected tumors and mounted onto polylysine coated slides. Before staining, tissue slides were sequentially deparaffinized and rehydrated using xylene solution and ethanol series. Heat-induced epitope retrieval was carried out in boiling Tris-EDTA buffer (pH 9.0) for 20 minutes, followed by thorough wash with 0.025% Triton X-100 buffer. Blocking was conducted in TBS buffer containing 10% goat serum and 1% BSA. Slides were incubated overnight in a humid 4°C chamber with 1:200 diluted rabbit anti-MBD3 antibody (Pierce) or 1:500 diluted rabbit anti-5hmC antibody (Active Motif). After blocking endogenous peroxidase with 0.3% H_2_O_2_, goat anti-rabbit IgG-HRP conjugates (Life technologies) were applied for 1 hour at room temperature. Color signal was developed by using AEC staining Kit (Sigma) and then the slides were sealed in mounting medium prior to imaging.

### Construction of lentiviruses and cell transduction

shRNAs were used for *in vivo* tumor formation assay and the sequences are provided in Table S1. The shRNAs were constructed into the pLent-U6-GFP-Puro plasmid. Then the psPAX2 and pMD2.G packaging plasmids were co-transfected with the pLent-U6-GFP-Puro/pLent-MBD3 shRNAs in HEK293ft cells for 48 hours according to manufacturer's instructions. Medium with virus was collected after 48 hours of transfection and concentrated by centrifuge. The titer of viruses was tested and then preserved in -80°C. U87 cells were infected with shRNA constructs for 48 hours and selected with puromycin (0.75 mg/ml). 5 × 10^6^ infected U87 cells (GFP/MBD3 shRNAs) were injected to the thalamus of mice (BALB/c-nu, body weight 18-22g, 1-month old). One month after the injection, the mice were sacrificed and the tumors were taken out for measurement. All animal handling and operations followed the NIH Guide for the Care and Use of Laboratory Animals.

### Clinical cohort design

A panel of primary glioma biopsy samples with characterized WHO grades and the control specimens of normal brain tissues from non-cancer pathologies were obtained from the Department of Neurosurgery in Xiangya Hospital of Central South University (Changsha, China). Informed consent was obtained from all involved patients. After matching the clinical properties, MBD3 and 5hmC levels, 14 glioma patients were enrolled for the follow-up cohort, and their basic information was listed in Table S2. The tumor size and location were identified by MRI (T1 post-contrast images of GBM are provided in Figure 8). All surgical operations achieved gross total resection followed by adjuvant standard radiotherapy and chemotherapy. After a pathologist confirmed the glioma grade according to the 2007 WHO Classification of Tumors of the Central Nervous System, the tumor DNA and RNA were immediately extracted from the surgically resected tissues and part of the tumor was paraffin embedded. A 60-month follow-up was conducted: PFS is defined as the living time before tumor recurrence; OS is herein defined as the time before clinical death or last follow-up.

## SUPPLEMENTARY FIGURES AND TABLES


