# Supplementary material for: Regulatory landscape and clinical implication of MBD3 in human malignant glioma
Source: Oncotarget. 2016 Nov 7;7(49):81698–714. doi: 10.18632/oncotarget.13173 (PMC5340251; doi:10.18632/oncotarget.13173)
Supplement: Supplementary file 1 [file oncotarget-07-81698-s001.pdf]

Regulatory landscape and clinical implication of MBD3 in human malignant glioma

SUPPLEMENTARY MATERIALS AND METHODS

Fluorescence correlation spectroscopy (FCS)

FCS was conducted with a scanning confocal time-resolved microscope (Microtime, PicoQuant GmbH) to test the chromatin binding states of MBD3 in SF767 cells. Using autocorrelation function, the diffusion profile of fluorescent molecules can be calculated by:

$$G(\tau) = \frac{1}{\langle N \rangle} \cdot \sum_{i=1}^{n} f_i \cdot \frac{1}{1+\frac{\tau}{\tau_{Di}}} \cdot \frac{1}{\sqrt{1+\frac{1}{\kappa^2}\cdot\frac{\tau}{\tau_{Di}}}}$$

where $\langle N \rangle$ is the average number of diffusing molecules inside the detection volume, τ is the lag time between two measured fluorescence intensities, and τ_{Di} is the diffusion time for the ith component of the molecules. For most proteins in living cells, i can be set to 2 to differentiate the bound and unbound status of the molecules, as well as the respective proportion f_i. The MBD3-GFP plasmid was transfected following the previously optimized protocol and the phototoxicity was carefully controlled in FCS measurements [1].

In FCS data collection and analysis, only cells with a MBD3-GFP expression level at 80-100 nM were selected. In parallel to be fitted with the least-square algorithm provided in the SymPhoTime software (PicoQuant GmbH), autocorrelation functions were fitted by a Maximum Entropy Method (MEMFCS) as validation [2, 3]. In a general comparison, the percentage of chromatin-bound MBD3-GFP in glioma cells was found to be 50-70% while the bound component in noncancerous cells was less than 30%.

Colorimetric immunoassay for 5mC

DNA from control and siRNA-treated SF767 cells was extracted and purified with DNeasy Blood & Tissue Kit (Qiagen), according to the manufacturer's instruction. Global DNA methylation was quantified by MethylFlash Methylated DNA Quantification Kit (Epigentek). In brief, 100 ng of DNA was deposited to the bottom of the assay well followed by incubation with capture antibody and detection antibody, sequentially. The developed colorimetric signals were normalized over the standard input DNA to give out the global methylation percentage in samples.

MSP for CIITA promoter IV

The methylation status for the promoter IV region of CIITA gene was determined using MSP. After DNA extraction, bisulfite conversion was performed with EZ DNA Methylation-Lightning Kit (Zymo Research). PCR primer design and reaction condition followed previous research [4]. The amplicons by unmethylated primers and methylated primers were run through 3% agarose gel pre-stained with GelRed dye (Biotium). The gel band intensity was analyzed with the ImageJ software (http://imagej.nih.gov/ij).

Flow cytometry

Flow cytometry was applied to determine cell cycle progression. In general, cells were fixed with 70% ethanol and then stained with 20 µg/ml propidium iodide (PI) solution containing 0.1% Triton X-100 and 200 µg/ml RNase A, referring to the established protocol [5]. A FC500 MPL system (Beckman Coulter) was used to quantify the single-cell PI intensity from at least 15,000 cells. The generated cell cycle histograms were analyzed with Flowjo software.

Immunofluorescence staining

Cells were first fixed with 4% paraformaldehyde for 15 minutes and then permeabilized with 0.4% Triton X-100 for 30 minutes. For 5mC staining, cells were additionally treated with 1 N HCl at 37°C for 30 minutes. Blocking was conducted with fresh PBS buffer containing 5% goat serum and 0.3% Triton X-100. Primary antibodies used in this study include mouse anti-5mC antibody (Eurogentec) and rabbit anti-MBD3 antibody (Pierce). Cells were incubated with primary antibody at 4°C overnight and then with secondary antibodies: AlexaFluor-546 $F(ab')_2$ fragment of Goat anti-Mouse IgG and AlexaFluor-488 $F(ab')_2$ fragment of Goat anti-Rabbit IgG, respectively. Confocal images were taken with an Olympus IX71 inverted microscope fitted with diode lasers.

Therapeutic strategy for enrolled patients

All patients received surgical resection to the extent feasible, followed by radiotherapy and chemotherapy which were given around 2~4 weeks after the surgical removal. Radiotherapy was given with a dose of 1.8 Gy/d and a total dose of 50-56 Gy which includes 30-36 Gy for brain and spine, and 20 Gy for the original tumor site as local boost. Chemotherapy mainly involved oral administration of temozolomide. One treatment cycle of chemotherapy was a successive dosage of 150 mg/m^2/d for 5 days within every 4 weeks. 8 cycles were performed in total. All the surgeries were performed in the Department of Neurosurgery, Xiangya Hospital, and the radiotherapy and chemotherapy were conducted in the Department of Oncology at Xiangya Hospital, Changsha City, China.

REFERENCES

1. Cui Y, Irudayaraj J. Dissecting the behavior and function of MBD3 in DNA methylation homeostasis by single-molecule spectroscopy and microscopy. Nucleic Acids Res. 2015; 43:3046-3055.
2. Nag S, Chen J, Irudayaraj J, Maiti S. Measurement of the attachment and assembly of small amyloid-beta oligomers on live cell membranes at physiological concentrations using single-molecule tools. Biophys J. 2010; 99:1969-1975.
3. Cui Y, Cho IH, Chowdhury B, Irudayaraj J. Real-time dynamics of methyl-CpG-binding domain protein 3 and its role in DNA demethylation by fluorescence correlation spectroscopy. Epigenetics. 2013; 8:1089-1100.
4. Garrity-Park MM, Loftus EV, Jr., Sandborn WJ, Bryant SC, Smyrk TC. MHC Class II alleles in ulcerative colitis-associated colorectal cancer. Gut. 2009; 58:1226-1233.
5. Juan G, Traganos F, Darzynkiewicz Z. Methods to identify mitotic cells by flow cytometry. Methods Cell Biol. 2001; 63:343-354.

SUPPLEMENTARY FIGURES AND TABLES

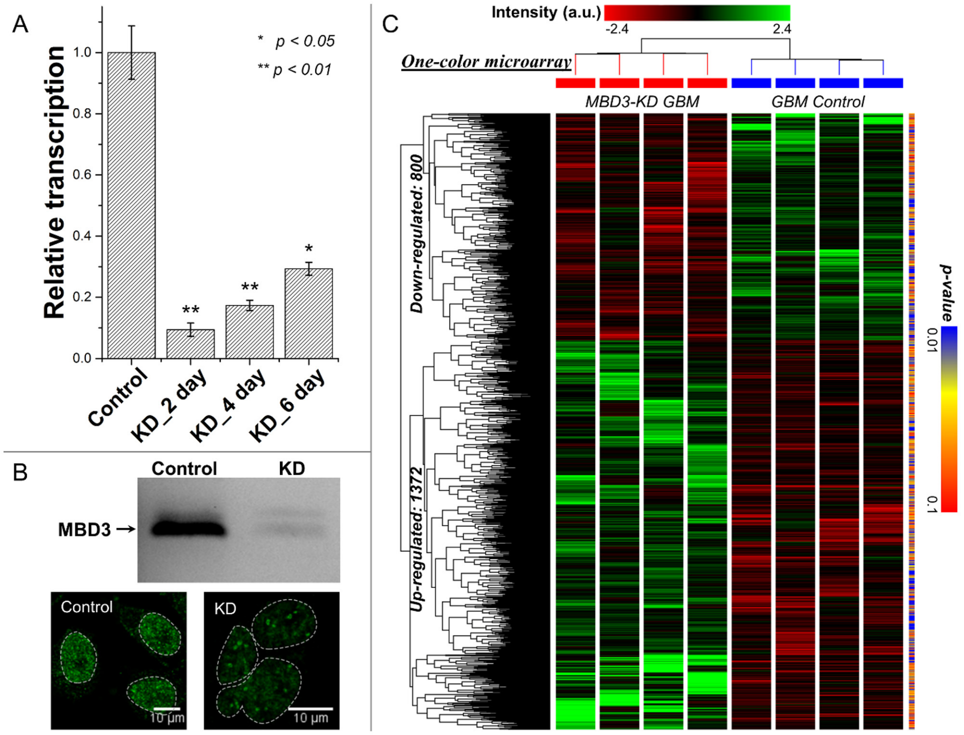

Supplementary Figure S1: Profile of the genome-wide transcriptome regulated by MBD3 in human GBM cells. Knockdown efficiency by MBD3-siRNA was validated by **A.** qRT-PCR (up to Day 6, n = 4), **B.** Western blot and immunofluorescence staining (at Day 4). **C.** Upon knockdown of MBD3, the changes in transcriptome were quantified by Agilent microarray.

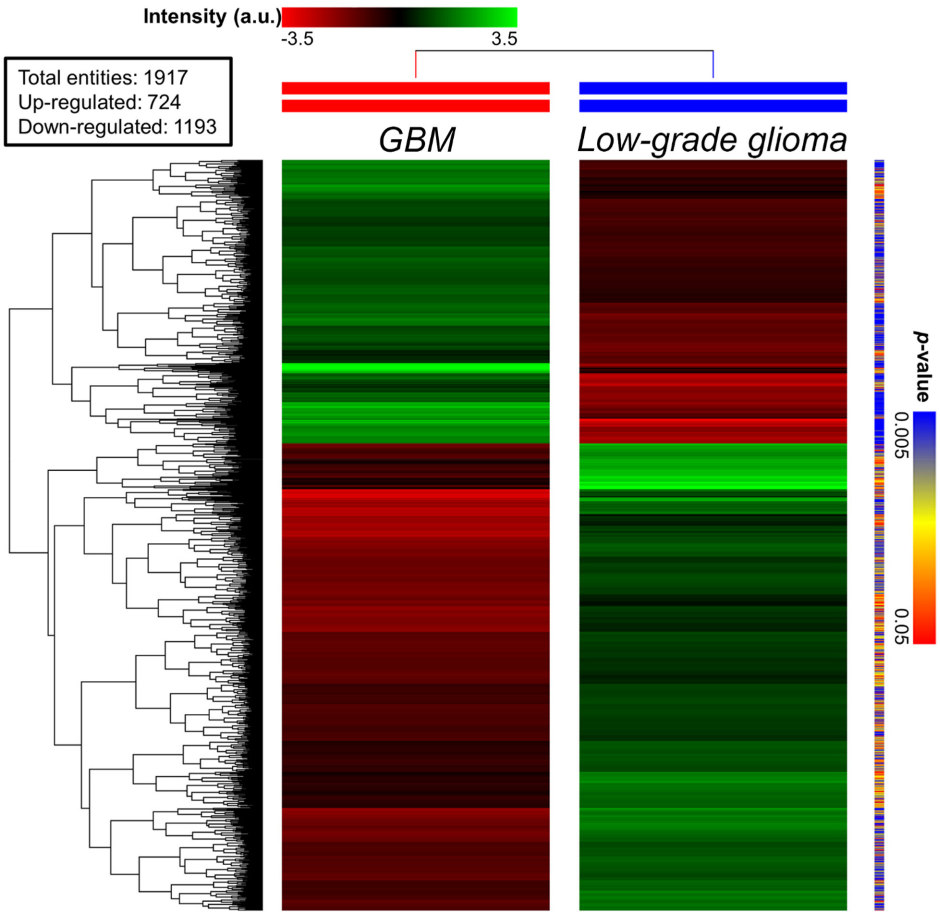

Supplementary Figure S2: DEGs between GBM and low-grade glioma were profiled (averaged from 8 patients).

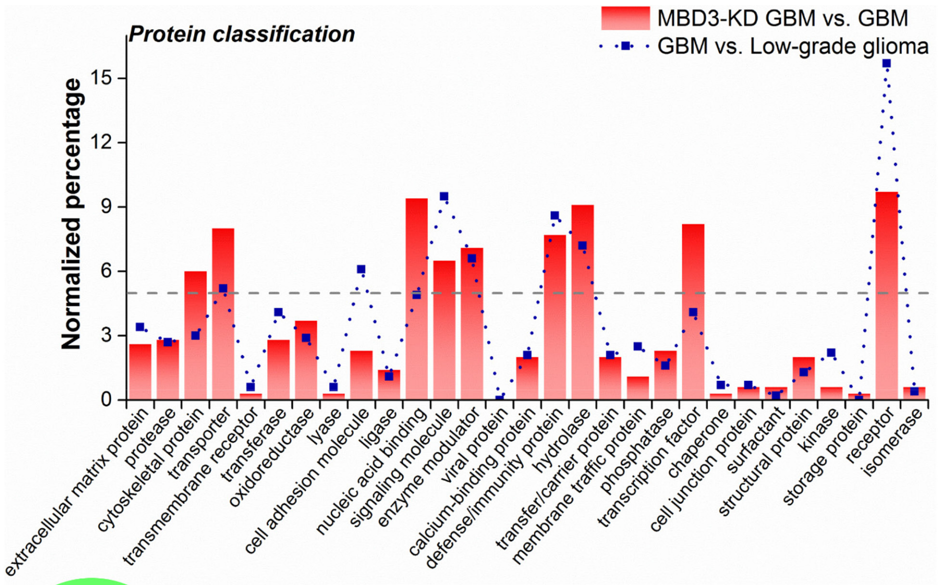

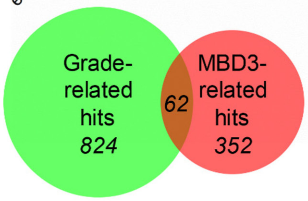

GO Analysis: MBD3-KD vs. Control	Gene counts	p-value
MHC Ⅱ protein complex	11	9.77E-17
Vacuolar membrane	17	2.55E-09
Cellular response to cytokine stimulus	21	1.61E-07
Lysosomal membrane	13	2.97E-07
Membrane part	147	1.13E-05

Supplementary Figure S3: Protein classification and GO analysis for the down-regulated genes in MBD3-KD GBM cells. In parallel, the differentiated expressed proteins between GBM and low-grade glioma was plotted as comparison (dash line). The number of overlapped protein hits during classification is presented in the pie chart. The top 5 matched GO terms mediated by MBD3 is presented in the table.

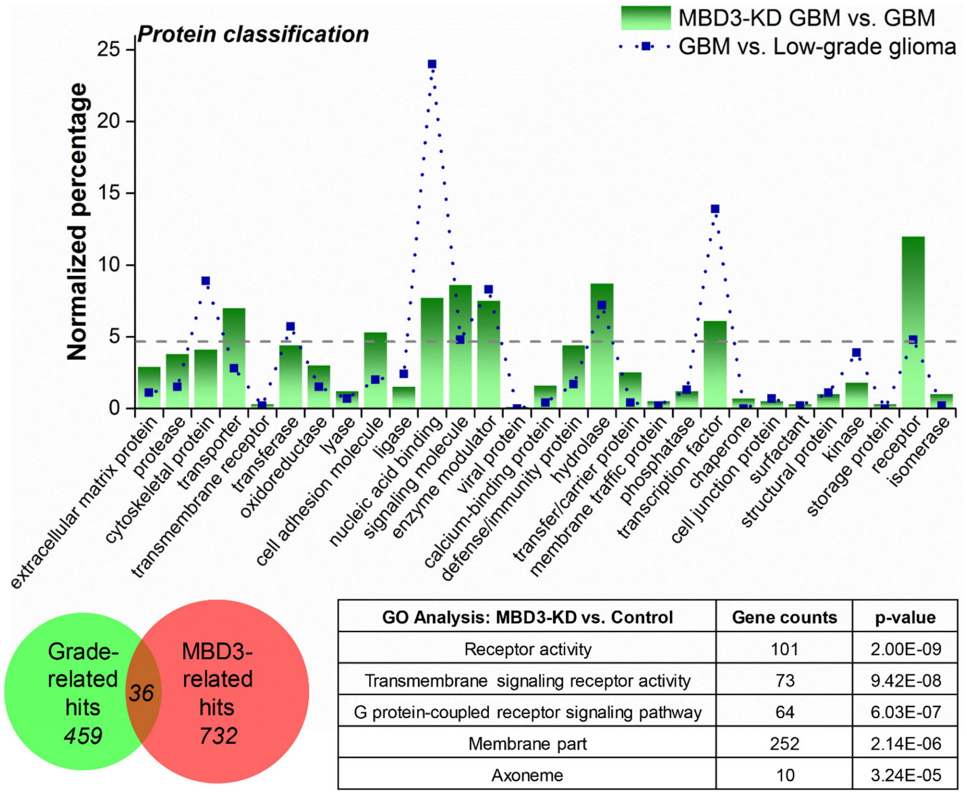

GO Analysis: MBD3-KD vs. Control	Gene counts	p-value
Receptor activity	101	2.00E-09
Transmembrane signaling receptor activity	73	9.42E-08
G protein-coupled receptor signaling pathway	64	6.03E-07
Membrane part	252	2.14E-06
Axoneme	10	3.24E-05

Supplementary Figure S4: Protein classification and GO analysis for the up-regulated genes in MBD3-KD GBM cells.

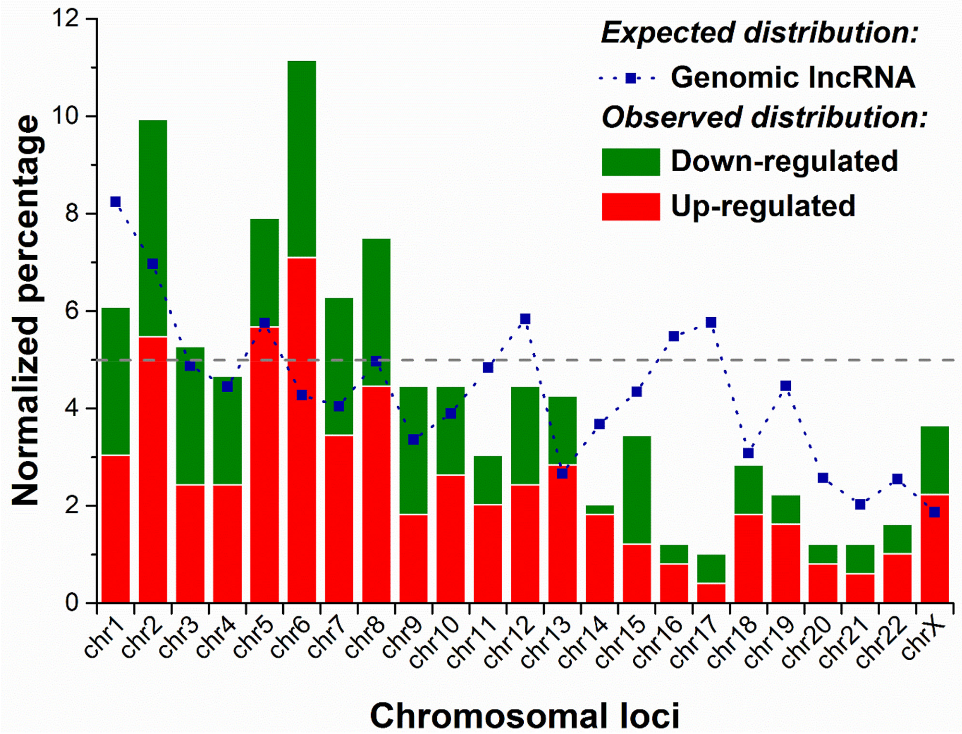

Supplementary Figure S5: Chromosomal distribution of the MBD3-regulated lncRNAs. The expected distribution of lncRNAs was retrieved based on the data in the Ensembl repository (http://useast.ensembl.org).

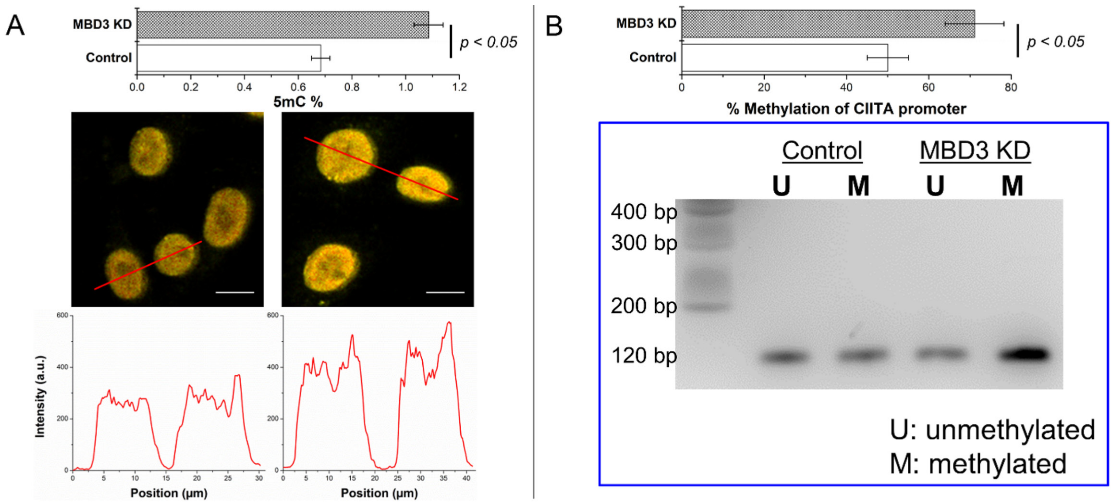

Supplementary Figure S6: MBD3 regulates the expression of the CIITA gene through promoter DNA methylation. **A.** Global hypermethylation induced by depletion of MBD3 was assessed by immunoassay (upper panel, n = 3) and immunofluorescence (lower panel). (Scale bars: 10 μm) **B.** Increase in the methylation level of the CIITA gene promoter was validated by MSP (n = 3).

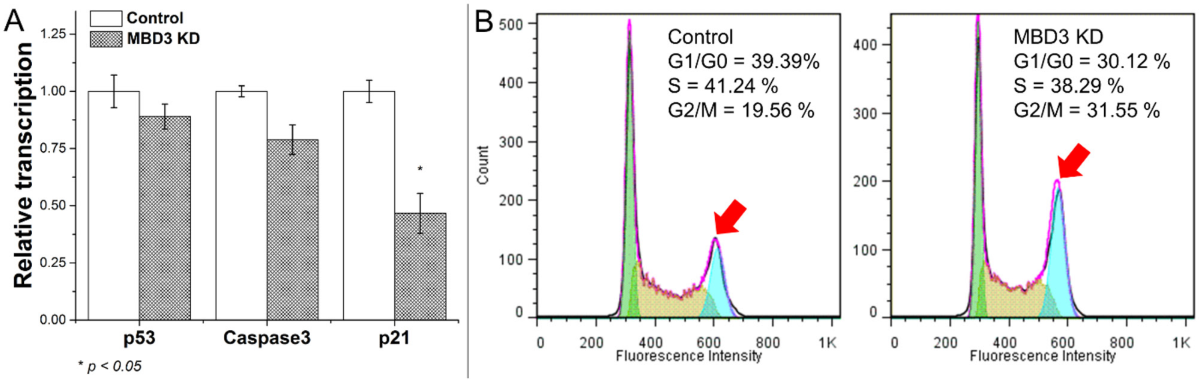

Supplementary Figure S7: Up-regulated miRNA-17-92 reduces p21 and disturbs cell cycle progression. A. Transcripts of p53, caspase3 and p21 were quantified under the MBD3 knockdown-caused overexpression of oncomiR-1 (n = 4). **B.** Cell cycle progression was analyzed by PI staining and flow cytometry.

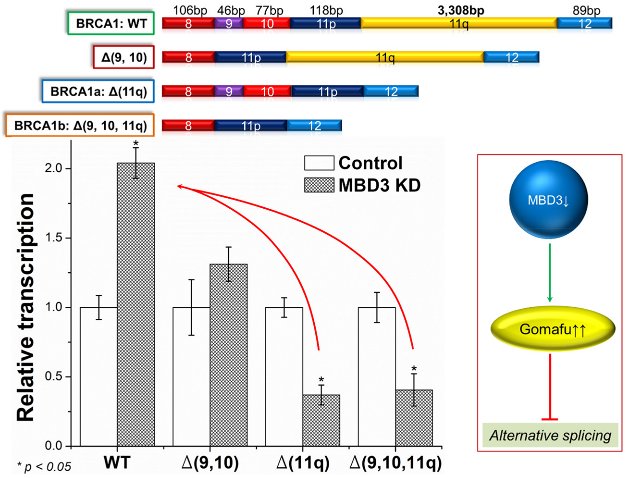

Supplementary Figure S8: Depletion of MBD3 deregulates the alternative splicing of BRCA1 through an increased Gomafu lncRNA. BRCA1 is a tumor suppressor gene experiencing active alternative splicing and Gomafu is a recently identified splicing inhibitor. Here the full length BRCA1 and its three common splice variants were quantified by RT-PCR (n = 4). Suppression of MBD3 leads to a significant increase of Gomafu lncRNA (> 12 folds from microarray), while the two BRCA1 variants supposed to splice out exon.11q drastically decrease.

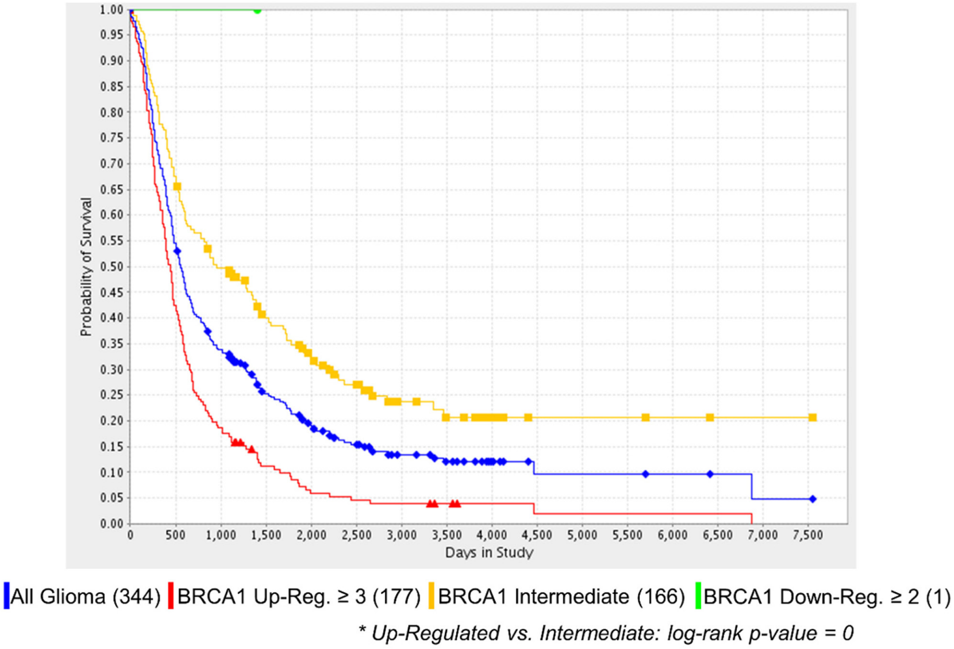

Supplementary Figure S9: Clinical significance of BRCA1 expression in the survival of glioma patients.

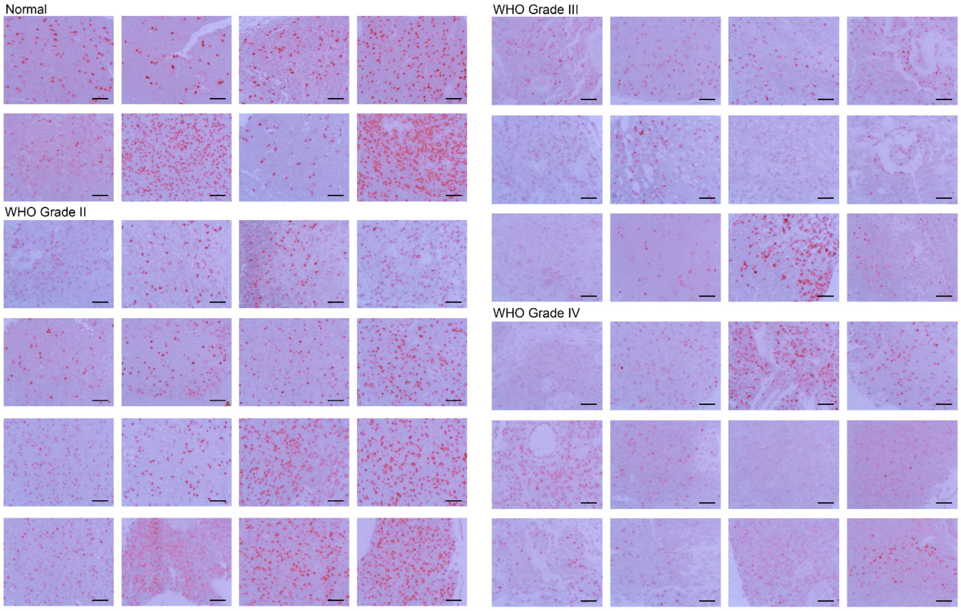

Supplementary Figure S10: Immunohistochemistry staining for MBD3 protein in glioma biopsy tissues. (Scale bars: 100 μm)

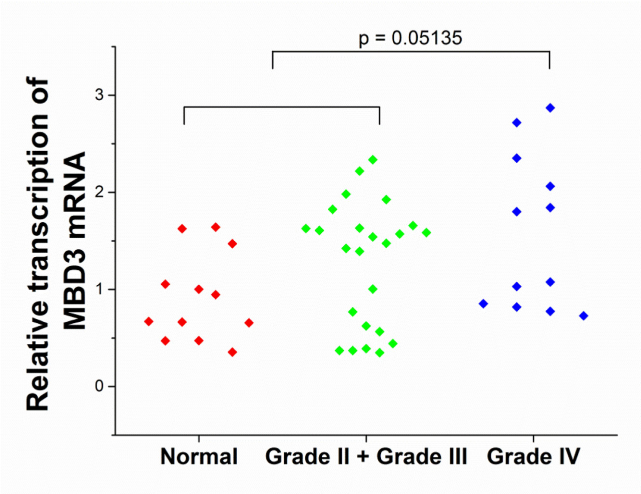

Supplementary Figure S11: Transcription of MBD3 in normal brain tissue and different grades of glioma.

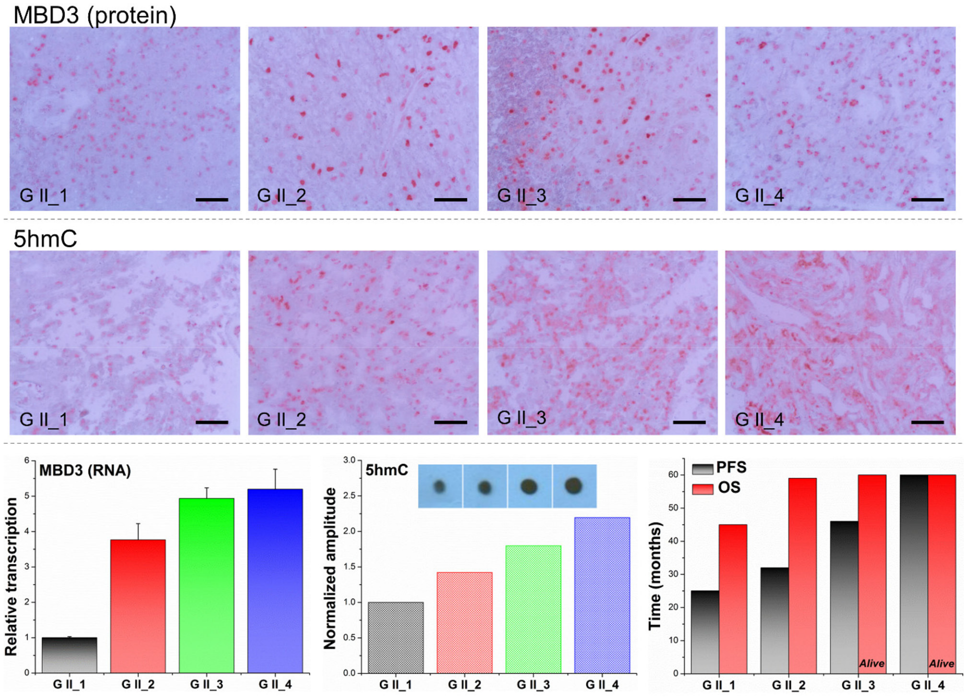

Supplementary Figure S12: MBD3 expression, 5hmC level, and survival of recruited Grade II patients. 4 patients with correlated MBD3 and 5hmC content were followed up after surgery. Better survival was observed in patients with higher MBD3 and 5hmC in their tissue biopsies. (Scale bars: 100 µm)

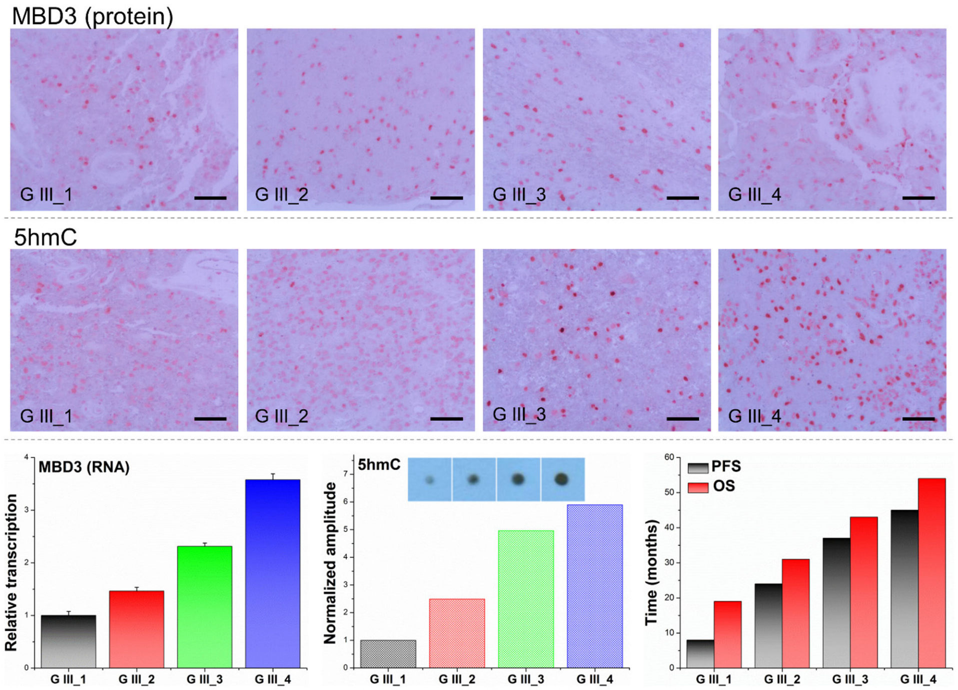

Supplementary Figure S13: MBD3 expression, 5hmC level, and survival of recruited Grade III patients. 4 patients with correlated MBD3 and 5hmC content were followed up after surgery. Similarly, better survival was observed in patients with higher MBD3 and 5hmC in their tissue biopsies. (Scale bars: 100 µm)

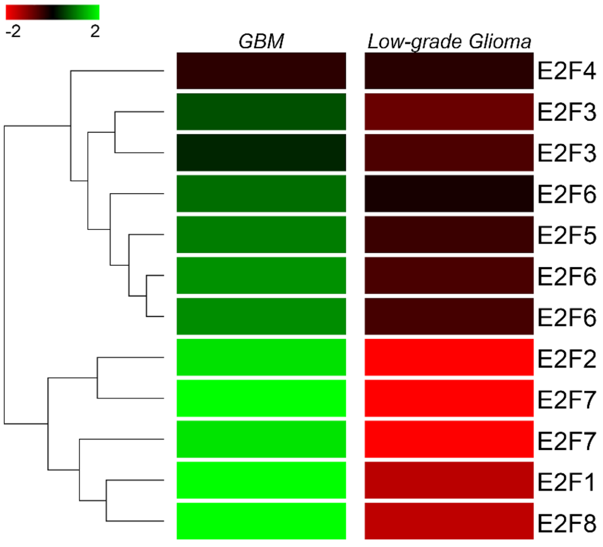

Gene	*Fold-increase*
E2F1	7.77
E2F2	16.04
E2F3	1.86
E2F5	2.11
E2F6	2.17
E2F7	10.84
E2F8	10.54

Supplementary Figure S14: Differential expression of E2F family proteins between GBM and low-grade glioma.

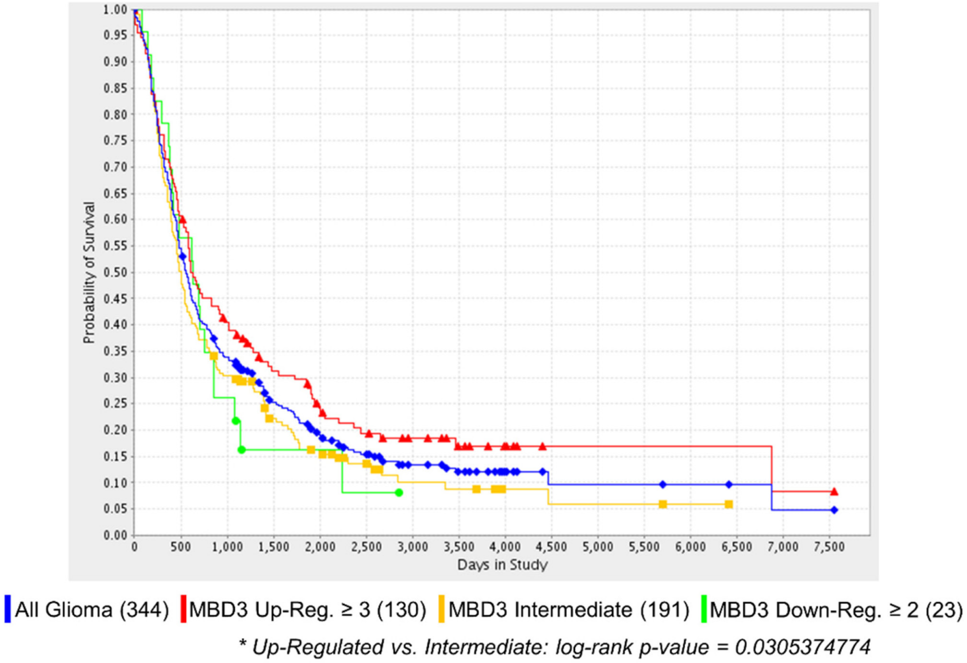

Supplementary Figure S15: Clinical implication of MBD3 in the survival of glioma patients.

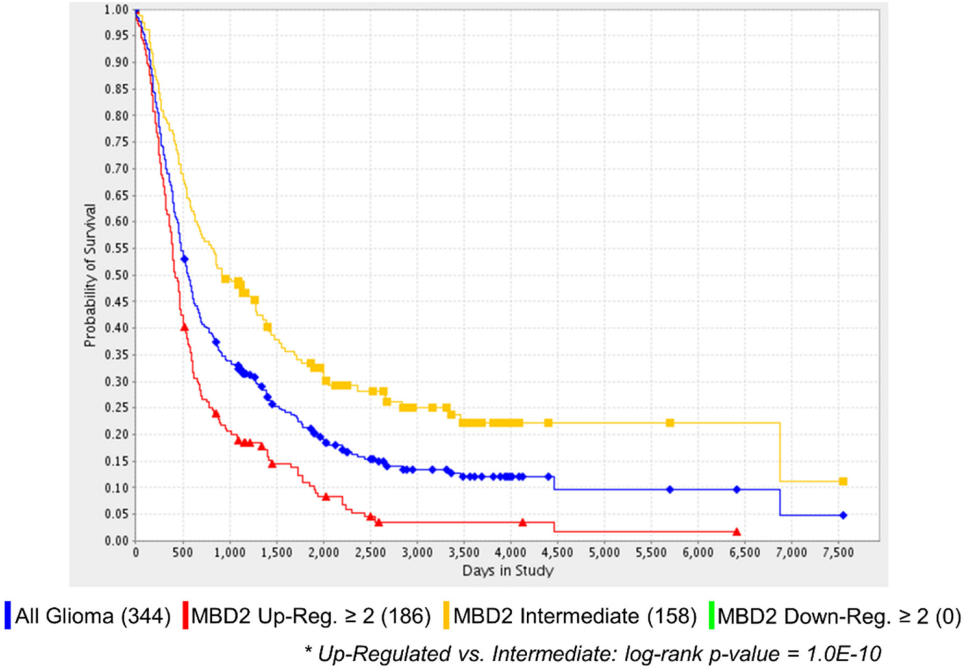

Supplementary Figure S16: Clinical implication of MBD2 in the survival of glioma patients.

Supplementary Table S1: Sequences of shRNAs used in *in vivo* experiments

1	GCAAGATGCTGATGAGCAAGATG
2	GGTGACCAAGATTACCAACCACC
3	GACATTGCTGAGGAGCTGGTCAA
4	GACATCAGGAAGCAGGAAGAGCT

Supplementary Table S2: Information of patients recruited for the clinical follow-up

Patient #	Gender	Age	Symptoms	Tumor location
G II_1	Female	37	Seizure	L. frontal
G II_2	Male	36	Headache	R. temporal
G II_3	Female	46	Headache; Visual impairment	L. frontal & temporal
G II_4	Male	42	Seizure; L. limb weakness	R. frontal
G III_1	Male	44	R. limb weakness & numbness	L. frontal & parietal
G III_2	Male	48	Nausea & vomiting	L. temporal
G III_3	Male	62	Visual impairment	R. frontal
G III_4	Male	57	Poor memory; Headache	L. insula
GBM_1	Male	37	Seizure; R. limb weakness & numbness	L. frontal & parietal
GBM_2	Male	58	Headache; Poor memory	R. temporal & insula
GBM_3	Male	27	Headache; Dizziness; Seizure	L. temporal
GBM_4	Male	46	Headache; Visual impairment; Poor memory	L. temporal
GBM_5	Female	55	Headache	L. temporal
GBM_6	Female	26	R. limb weakness	R. frontal & parietal

Supplementary Table S3: RT-PCR primers used in this study

GAPDH	(F)CAGCCTCAAGATCATCAGCA<br>(R)TGTGGTCATGAGTCCTTCCA
p53	(F)CCCCTCCTGGCCCCTGTCATCTTC<br>(R)GCAGCGCCTCACAACCTCCGTCAT
p21	(F)GAGGCCGGGATGAGTTGGGAGGAG<br>(R)CAGCCGGCGTTTGGAGTGGTAGAA
Caspase3	(F)ATGATGACATGGCGTGTCATAA<br>(R)AAGGAAAAGGACTCAAATTCTGTTG
MBD3	(F)GGCCACAGGGATGTCTTTTA<br>(R)TTGACCTGGTTGGAGGAGTCG
BRCA1 WT	(F)CAAGGAACCAGGGATGAAATCAG<br>(R)ATGGCTCCACATGCAAGTTTG
BRCA1 Δ(9,10)	(F)CTACATTGAATTGGCTGCTTGTGA<br>(R)CATGCTGTAATGAGCTGGCATGA
BRCA1 Δ(11q)	(F)TTACAAATCACCCCTCAAGGAACC<br>(R)CACACCCAGATGCTGCTTCAC
BRCA1 Δ(9,10,11q)	(F)CTACATTGAATTGGCTGCTTGTGA<br>(R)CACACCCAGATGCTGCTTCAC
